# Interpersonal Psychotherapy for Depression in Parkinson’s Disease: A Feasibility Study

**DOI:** 10.1177/08919887221090220

**Published:** 2022-04-21

**Authors:** Diana Koszycki, Monica Taljaard, Cary Kogan, Jacques Bradwejn, David Grimes

**Affiliations:** 1 6363 University of Ottawa, Ottawa, ON, Canada; 2 Institut du savoir Montfort, 153195 Montfort Hospital, Ottawa, ON, Canada; 3 151181 University of Ottawa Brain and Mind Research Institute, Ottawa, ON, Canada; 4 12365 Ottawa Hospital Research Institute, Ottawa, ON, Canada; 5 Université de Montreal, QC, Canada

**Keywords:** Parkinson’s disease, depression, psychotherapy, interpersonal psychotherapy, IPT, feasibility study

## Abstract

Individuals living with Parkinson’s disease (PD) experience interpersonal stressors that contribute to depressive risk. Interpersonal psychotherapy (IPT) emphasizes the bidirectional relationship between interpersonal stressors and mood may therefore be a suitable treatment for PD-depression. The primary aim of this study was to evaluate the feasibility of delivering 12 sessions of IPT to depressed PD patients and explore the need for modifications. A secondary aim was to obtain descriptive information about efficacy outcomes. The study used a pre-post design without a comparison group. Participants were 12 PD patients with a major depressive disorder. IPT was well accepted and tolerated by patients and required minimal modifications. Compliance with session attendance and completion of study questionnaires were excellent and treatment satisfaction was high. Depression scores declined from baseline to endpoint, with 7 patients meeting criteria for remission at endpoint. Findings are encouraging and a larger randomized controlled trial is currently underway to ascertain if IPT is an efficacious treatment for PD-depression.

## Introduction

Depression is a common non-motor complication of Parkinson’s disease (PD) with an estimated 35% of patients reporting clinically significant depressive symptoms.^1^ Depression can significantly affect motor and cognitive symptoms of PD and is considered the most significant predictor of impaired quality of life and more important than severity of motor symptoms.^2-5^ Pharmacotherapy is often the first choice of treatment for PD-depression because of an emphasis on biological factors in the pathophysiology of depressive symptoms in PD.^6^ While effective, medications with an antidepressant effect are not without limitations and may be associated with unpleasant side effects and risk for potential interactions with other medications.^7^ There is also greater risk for poor response to pharmacotherapy if psychosocial factors that contribute to or exacerbate depression are not addressed.^8^ This is important to note as depressed PD patients have been reported to attribute the cause of their depressive symptoms to psychosocial rather than to biological factors and have a more favorable view towards psychotherapy than pharmacotherapy.^9^ Satisfaction with treatment for mental health problems is also higher when PD patients receive a combination of medication plus psychotherapy vs medication alone.^10^ Considering that psychological factors confer risk for depression over and beyond motor symptoms,^11^ psychotherapy is an important therapeutic option for PD-depression, especially for patients who desire a nonpharmacological approach or for those who may benefit from combined pharmacotherapy and psychotherapy.

Among the depression-focused psychotherapies, interpersonal psychotherapy (IPT) is well established as an effective first-line treatment for depression across the lifespan.^12^ IPT is a time-limited (12–16 sessions) intervention that was developed in the 1970s by Klerman, Weissman and colleagues as an acute treatment for major depressive disorder (MDD).^13^ Since its development, over 133 randomized clinical trials have been published for different psychiatric disorders including 91 trials on depression.^14^ IPT recognizes that depression is a medical illness with an underlying biological component, making it easy to combine the therapy with antidepressant medication if indicated. IPT’s medical model of depression helps patients to reframe attribution of causality (i.e., depression is an illness and not the patients’ fault), but also emphasizes their responsibility to get well and comply with treatment. IPT is grounded in interpersonal and social attachment theory and purports that irrespective of cause, most clinical depressions occur in an interpersonal context.^15^ IPT emphasizes the bidirectional relationship between interpersonal stressors and mood and links the onset, maintenance and recurrence of depressive symptoms to four interpersonal problem areas: *complicated bereavement* following the death of a close attachment, *role transitions*, which involve difficulty adjusting to life transitions or losses, *role disputes*, which involve conflicts with significant others, and *interpersonal deficits*, which involve social isolation and lack of social supports.^15^ The goal of IPT is to resolve these interpersonal stressors (e.g., facilitate grieving, resolve interpersonal conflicts and transitions) and strengthen social supports.

IPT’s focus on interpersonal stressors makes it a suitable intervention for depression comorbid with medical conditions.^16^ Individuals living with PD experience interpersonal stressors that contribute to increased depressive risk, including relationship discord,^4^ attachment insecurity,^17^ and loss of valued activities, social roles, and social connectedness brought about by the illness.^18,19^ Social isolation and loss of network supports and attachments due to death, relocation, or other circumstances may also contribute to depressive risk,^20^ especially among older PD patients. IPT’s emphasis on building and strengthening supportive relationships has relevance for PD because the disorder and its treatment are chronic stressors and adequate social supports can have stress-buffering effects.^21^ PD patients with limited or poor social supports have been found to be at increased risk for depression, experience greater impairment due to motor symptoms, and have poorer quality of life.^22^

Although IPT and cognitive behavior therapy (CBT) are well established psychological treatment options for depression^12^ there is a paucity of research on the efficacy of these interventions for PD-depression. There is encouraging evidence that CBT adapted for PD may be an efficacious antidepressant treatment, with randomized trials demonstrating superiority of CBT relative to clinical monitoring,^23^ treatment as usual,^24^ and waitlist control.^25^ However, we are unaware of any published research on the acceptability and potential efficacy of IPT. The primary aim of this study was to evaluate the feasibility of delivering IPT for PD-depression. A secondary aim was to obtain descriptive information about the effects of IPT on depressive symptoms, quality of life, and interpersonal relationships. An exploratory aim was to describe changes in outcomes up to 3 months post-treatment.

## Methods

### Study Design

The study used a pre-post design without a comparison group and results were used to inform the design of a fully powered randomized controlled trial.

### Participants

Participants were recruited from a large movement disorders clinic at a University of Ottawa teaching hospital. A sample size of 15 was considered sufficient to determine feasibility.^26^ The study was approved by the institutional review board and participants provided written informed consent. To be included in the study, participants had to have idiopathic PD with Hoehn and Yahr stages I–III,^27^ be on a stable dose of dopaminergic replacement, or if not currently on dopamine replacement therapy, assessed as not likely to require therapy for at least 12 weeks, live independently at home or a retirement facility, meet diagnostic criteria for MDD confirmed by a structured clinical interview,^28^ and obtain a score ≥14 on the 17-item Hamilton Depression Rating Scale (HAM-D)^29^ at the screen and baseline visit. A HAM-D score of 14 has been suggested as an optimal cut-off score to discriminate between PD patients with and without a depressive disorder.^30^

Exclusion criteria included a history of substance use disorders in the last 12 months, a lifetime history of psychosis or bipolar disorder, or high suicide risk. Other psychiatric disorders were allowed so long as MDD was the primary disorder. Patients with cognitive impairment (score ≤24 on the Mini Mental Status Exam),^31^ presence of other significant neurological problems and unstable comorbid medical conditions, current treatment with any form of psychotherapy, and poor hearing acuity that could affect communication in therapy were also excluded. Concurrent use of medications with antidepressant, anxiolytic or hypnotic effects or herbal products with psychoactive properties was allowed if the medication type and dose had remained stable for 8 weeks prior to starting psychotherapy and there was no change in medication during acute treatment with IPT. The use of concomitant medication was monitored during the scheduled assessments. Concomitant psychotherapy was proscribed.

### Interpersonal Psychotherapy

Participants received 12, 1-hour therapy sessions by a trained IPT therapist with experience working with medically ill patients including those with PD. IPT has three phases (beginning, intermediate, termination), with each phase involving specific tasks and strategies. In the beginning phase the therapist explains depression as a medical illness that is treatable and not the patient’s fault, reviews the patient’s current interpersonal relationships, and identifies one, or at the most two, interpersonal problem areas that are most salient to the patient’s depressive symptoms. The middle phase of therapy focuses on resolving interpersonal stressors and strengthening social supports. The final phase of therapy focuses on consolidating treatment gains, relapse prevention, and assessing the need for continued therapy.

The IPT manual developed by Weissman et al^32^ was used. Sessions were delivered on a face-to-face basis but 2–3 telephone sessions were allowed if attendance was difficult due to poor health, transportation problems, or caregiver burden. Although IPT is designed as a one-to-one therapy, occasional joint sessions with close members of the patient’s social network are allowed.^32^ The present study determined if it was feasible to include a primary family support person in up to three joint sessions. These sessions were intended to educate the support person about depression in the context of PD, explore ways that they can support the patient, and discuss challenges they may face as a primary support person. Slight modifications were made to meet the needs of PD patients. This included informing patients that they had two medical illnesses (depression and PD) and providing education about both depression and PD and their interaction. Because patients and their families often believe that feeling depressed is normal for someone living with PD, it was important to emphasize that depression is a serious condition that affects functioning and quality of life and that not all patients living with PD become clinically depressed.

### Measures

Feasibility outcomes included program completion, completion of post-intervention questionnaires, patient satisfaction, and participation of a primary family support person in up to three joint sessions. This study aimed to achieve a 75% program completion rate, a loss of post-treatment data not exceeding 25%, and a 75% participation rate of a primary family support person. Patient satisfaction was assessed with Client Satisfaction Questionnaire (CSQ), an 8-item scale that assesses consumer satisfaction with mental health services. Total scores range from 0 to 32, with higher scores indicating greater treatment satisfaction. The CSQ demonstrates solid psychometric properties including adequate internal consistency.^33^

The primary efficacy outcome was the 17-item HAM-D.^29^ The HAM-D is a widely used primary outcome in clinical trials of depression and is considered a reliable measure of depression in PD patients.^34^ The scale was administered by telephone by the same clinician who was not involved in the treatment of participants. The clinician had extensive experience conducting interviews with the HAM-D for clinical trials using the grid scoring convention.^35^ Additional outcomes included response and remission rates and self-reported depression, health-related quality of life, and interpersonal supports. Response was defined as a 50% reduction from baseline in HAM-D scores and remission as a HAM-D score ≤6. Self-reported depression was assessed with the Beck Depression Inventory-II (BDI-II),^36^ a 21-item scale that assesses severity of depressive symptoms over a 2-week period. Items are rated on a 0 to 3 scale with scores ranging from 0 to 63. The BDI-II has excellent psychometric properties and is considered a reliable measure of depression in PD patients.^34^ A 17.5% reduction in BDI-II scores has been suggested as a minimally clinically important difference based on receiver operator characteristics (ROC).^37^

Quality of life was measured with the Parkinson’s Disease Questionnaire-39 (PDQ-39), a 39-item scale specifically developed to assess health-related quality of life in PD.^37,38^ It produces eight distinct dimensions (mobility, activities of daily living, emotional well-being, stigma, social support, cognitions, communication, and bodily discomfort) and one summary index (PDQ 39-SI). Items are rated on a 5-point Likert scale with scores ranging from 0 (no difficulty) to 100 (maximum difficulty). Higher PDQ scores reflect poorer quality of life. The scale has good psychometric properties, including construct validity and test-retest reliability, and has demonstrated responsiveness to change in patient status in longitudinal studies.^39^ A 4.72 reduction in PDQ-SI scores has been suggested as a minimally clinically important difference based on ROC.^40^ The Interpersonal Relationships Inventory (IPRI)^41^ is a 39-item scale that assesses three domains of interpersonal relationships: perceived availability support supports, reciprocity, and conflict. Standard means are calculated for each subscale by dividing the subscale total score with the number of subscale items. Standard means range from 1 to 5, with higher scores indicating higher levels of perceived social supports, reciprocity, and conflict. The scale has good psychometric properties.

### Statistical Analysis

Feasibility outcomes were analyzed descriptively (mean and standard deviation for continuous variables; frequency and proportion for categorical variables) using SPSS version 27 and presented graphically using line diagrams. Since this was a feasibility study, it was not designed to test the efficacy of IPT and no formal statistical analysis was therefore conducted on clinical measures; instead, only descriptive statistics are reported.^42^ Estimates obtained from these descriptive analyses (e.g., standard deviations) were used to inform sample size calculation for a future large trial.

## Results

### Patient Characteristics

**Figure A1.**
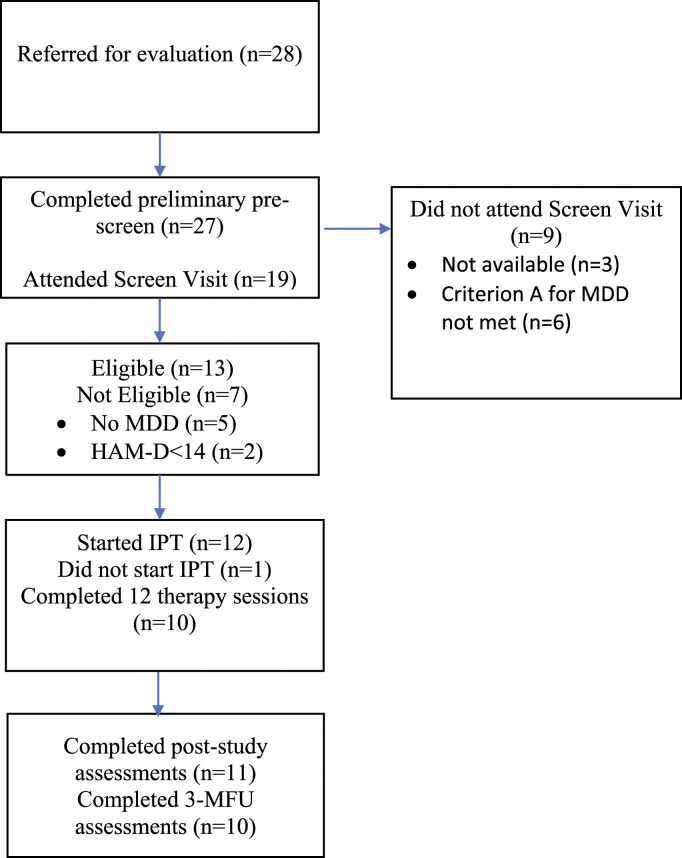
Flow of participants during the study.

Participants were recruited between February 2013 and January 2015. The flow of participants is displayed in Figure A1. Twenty-eight PD patients who were identified as being possibly depressed by clinic staff agreed to be contacted for the study. Twenty-seven completed preliminary screening by telephone. Of these, three were no longer available to participate in the study due to competing demands and five reported that they did not experience depressed mood nearly every day or had lost interest in most of their usual activities (Criterion A for MDD not met). Nineteen patients attended the face-to-face screening visit. Seven (35%) were not eligible because they did not meet criteria for MDD (n = 5) or had a HAM-D score <14 (n = 2). Of the 13 eligible patients enrolled in the study, one withdrew before starting therapy. Thus, 12 eligible patients started treatment (5 women, 7 men; mean (SD) age: 62.4 ± 7.90 years).

Most patients were white/Caucasian (n = 10; 83.3%) and married (n = 9; 75%). Two patients were employed, and the others were either retired (n = 6) or unable to work (n = 4). Mean age of onset of PD was 52.42 ± 15.26 years (median=58.0 years). The mean baseline HAM-D score of 17.08 (SD, 2.0) and BDI-II score of 24.33 (SD, 8.2) reflect a moderately severe depressed group. Six patients reported a prior history of depression, two had a history of suicide attempts, seven had received psychotherapy in the past, six had a comorbid anxiety disorder, and seven were taking an antidepressant medication at the time of the study. There was no change in use of psychotropic medication during the acute phase of treatment. At the 3-month follow-up, one patient reported that they were switched to another antidepressant medication, and another reported that they had initiated antidepressant medication.

### Feasibility Outcomes

Compliance with IPT was excellent. The mean (±SD) number of sessions attended was 11.00 ± 2.7, with 10 of the 12 patients (83%) who started treatment completing all 12 therapy sessions. One patient completed nine sessions and discontinued therapy because he no longer felt depressed. Another attended three sessions and was lost to follow-up. The primary efficacy measure HAM-D was administered to 11 patients (91.2%) at endpoint and 10 (83%) at the 3-month follow-up. Session 12 HAM-D assessment was completed by the therapist for one patient because the independent clinical evaluator was unavailable. Satisfaction with treatment was high (mean CSQ score = 27.7 ± 4.6; median=30.0), with all patients reporting that the therapy helped them with their problem and the majority (91%) reporting that most or almost most of their needs were met. Four patients who started treatment (33.3%) included a family support person in at least one therapy session; two included a spouse only, one included a spouse and child, and one included a sibling. The remaining patients either preferred not to include a family support person or did not have a family support person who could participate.

### Adverse Events

No adverse events occurred although in some patients, the therapist noted transient increases in tremor whenever negative emotional states associated with interpersonal stressors were explored in therapy. The therapist used this observation to highlight the link between psychological distress and worsening of motor symptoms and the importance of developing adaptive strategies to better manage interpersonal challenges.

### Additional Modifications to IPT

All therapy sessions were conducted in person. Most patients were able to tolerate the 1-hour sessions except for two older patients (>70 years) who requested that some sessions be shortened. Although IPT is typically delivered as 12 weekly sessions, flexibility in spacing appointments was included as a modification if needed. For example, one older patient requested that weekly sessions be changed to biweekly sessions because weekly sessions were too emotionally intense. Since many patients reported varying levels of anxiety, especially during the wearing-off of levodopa medication, the therapist recommended anxiety management techniques (e.g., abdominal breathing) that patients could practice at home. Encouraging compliance with activities that positively affect motor symptoms and improve functioning was also included in the psychoeducational component of therapy as loss of interest, decreased energy, passivity, and social withdrawal can compromise patients’ ability to engage in health promoting behaviors. For example, the therapist suggested that patients engage in regular physical activity that could be performed with others (e.g., going for regular walks with a family member or friend, joining a group exercise activity for PD). The interpersonal context was considered an important element because social supports can promote adherence to regular exercise in PD.^43^ Patients were not asked to monitor their physical activity as homework as this would be outside the scope of IPT. Rather, the therapist encouraged them to try their best and to seek out the support of others to implement and sustain regular physical activity.

### Interpersonal Problem Areas

The primary focus of therapy was role transition (n = 10) and interpersonal deficits (n = 1).

Five patients had a secondary interpersonal problem that was linked to their depression including role disputes (n = 3), role transition (n = 1), and interpersonal deficits (n = 1).

### Efficacy Outcomes

**Figure A2.**
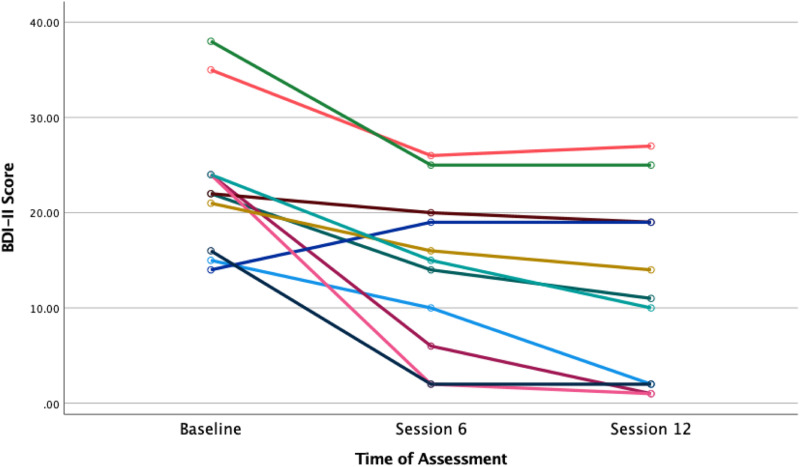
Spaghetti plot of BDI-II scores during acute IPT treatment. Note: Based on 11 participants who had complete data for each time point.

Table 1 displays descriptive statistics for clinician-and self-rated depressive symptoms. The mean (SD) difference in baseline to endpoint scores was −11.18(±4.8) points. Of the 11 patients who completed post-treatment HAM-D ratings, eight (72.3%) obtained a 50% reduction in HAM-D scores and seven (63.4%) met criteria for remission (i.e., HAM-D score ≤6). The mean (SD) difference in baseline to endpoint BDI-II scores was −11.27 ± 8.1, which corresponds to a 38% reduction in scores. Figure A2 displays a spaghetti plot of BDI-II scores during the acute phase of treatment. At the 3-month follow-up, HAM-D and BDI-II scores remained lower than baseline values (Table 1). Of the seven patients who achieved remission at endpoint, five remained in remission at the 3-month follow-up.

**Table 1.** Summary of Descriptive Statistics for Depression Ratings.

	Observed mean ± Standard Deviation (SD)					*Mean (±SD) difference from baseline at session 12*	*Mean (±SD) difference from baseline at 3-MFU*
*Outcome*	Baseline (N = 12)		*Session 6 (*N *= 11)*	*Session 12 (*N *= 11)*	*3-MFU (*N *= 10)*		
HAM-D	17.08 ± 2.0		9.18 ± 4.2	5.64 ± 4.9	5.10 ± 3.5	−11.45 ± 4.5	−11.60 ± 4.1
BDI-II	24.33 ± 8.2		14.09 ± 8.4	11.91 ± 9.7	10.60 ± 7.4	−11.27 ± 8.4	−11.40 ± 8.9

*Note:* HAM=D indicates 17-item Hamilton Depression Scale; BDI-II indicates Beck Depression Inventory-II. Lower scores indicate lower levels of depression.

Table 2 displays descriptive statistics for the PDQ-39 (Summary Index and dimensions) and the IPRI subscales at baseline, endpoint, and the 3-month follow-up. PDQ-39-SI scores decreased from baseline to endpoint, indicating improved perception of quality of life. As well, PDQ-39 dimensions decreased, with the largest decrease noted for well-being and stigma. Three-month follow-up scores were also lower than baseline scores. All three IPRI subscales changed in the expected direction.

**Table 2.** Summary of Descriptive Statistics for Measures of Health-Related Quality of Life and Interpersonal Relationships.

	*Observed mean ± Standard deviation*				*Mean difference from baseline at session 12*	*Mean difference from baseline at 3-MFU*
*Outcome*	*Baseline* N *= 12*	*Session 12* N *= 11*	*3-MFU* N *= 10*			
PDQ-39-SI	42.61 ± 18.1	22.86 ± 13.3	21.58 ± 10.6		−17.07 ± 21.1	−17.70 ± 17.0
PDQ-39 dimensions						
Mobility	38.87 ± 19.7	24.32 ± 18.9	23.25 ± 18.1		−10.27 ± 17.0	−12.30 ± 15.2
ADL	32.57 ± 27.5	17.80 ± 10.6	20.83 ± 14.7		−9.40 ± 21.3	−8.67 ± 16.5
Emotional wellness	61.75 ± 15.6	28.79 ± 21.2	26.25 ± 21.0		−31.00 ± 28.4	−30.35 ± 22.9
Stigma	68.19 ± 40.6	22.73 ± 30.1	20.83 ± 18.5		−39.54 ± 38.1	−37.66 ± 34.7
Social support	28.78 ± 20.2	13.64 ± 14.1	14.17 ± 17.1		−15.49 ± 22.8	−13.71 ± 24.2
Cognition	33.85 ± 18.6	21.02±15.4	20.63 ± 14.1		−11.93 ± 22.6	−13.13 ± 23.6
Communication	33.33 ± 25.9	21.97±23.1	25.00 ± 22.6		−6.82 ± 24.1	−6.67 ± 15.6
Bodily discomfort	46.54 ± 247	32.58 ± 24.4	21.67 ± 13.1		−12.12 ± 27.2	−19.17 ± 19.7
IPRI						
Support	3.79 ± 0.8	4.02 ± 0.9	4.01 ± 0.9		.28 ± 0.60	.27 ± 0.70
Reciprocity	3.69 ± 0.5	3.88 ± 0.6	3.79 ± 0.5		.25 ± 0.3	.16 ± 0.5
Conflict	2.30 ± 0.6	1.99 ± 0.7	1.99 ± 0.8		−.30 ± 0.5	−.30 ± 0.4

*Note:* PDQ-39-SI indicates Parkinson’s Disease Questionnaire Summary Index. ADL indicates Activity of Daily Living. IPRI indicates Interpersonal Relationships Inventory. Scores on the PDQ-39 range from 0 to 100, with lower scores reflecting better quality of life. Scores on the IPRI range from 1 to 5, with higher scores reflecting higher levels of interpersonal support, reciprocity, and conflict.

## Discussion

This preliminary open feasibility trial suggests that IPT is well accepted and tolerated by depressed PD patients. Compliance with session attendance and completion of study questionnaires were excellent and satisfaction with the intervention was high. The retention rate in this study is similar to other studies of IPT for medically ill patients^15,44-47^ and compares well to studies on the efficacy of CBT for PD-depression.^23,48^ The high retention rate is important as an adequate trial of psychotherapy is usually associated with better therapeutic response.^49^ Although we fell short of enrolling 15 patients in this study, a final sample of 12 patients (13 including the patient who did not start treatment) is still considered an appropriate sample size to assess feasibility.^26^

While some modifications to IPT were incorporated, the core elements of the therapy were well preserved, including the structure of therapy, therapist stance, and psychotherapeutic techniques. Only a third of patients who started treatment invited a family support person to at least one therapy session, which falls below the expected 75% participation rate. Most patients preferred not to include a family support person because they either did not want to burden them or were uncomfortable including them in a conjoint session. Thus, inclusion of a family support person should encouraged but not be a requirement for participation in future trials of IPT for PD-depression. While participation of a caregiver was an inclusion criterion in Dobkin et al’s CBT study for PD-depression,^23^ caregivers attended 3–4 psychoeducational sessions separately from the patient sessions and it is possible that separate sessions may be more appealing to some patients and caregivers than conjoint sessions. This should be explored in future research of IPT for PD-depression.

Examination of efficacy measures showed that depression severity decreased over time. The 11.18-point decrease from baseline to endpoint in HAM-D scores is similar to other uncontrolled studies of IPT for medically ill patients^16,50^ but somewhat higher than uncontrolled studies of CBT for PD-depression.^48,51^ Our response and remission rates are generally comparable to other studies of IPT for depressed medically ill patients^15,44,46^ as well as CBT for PD-depression.^23,48,51^ Further, the mean reduction of 38% in BDI-II scores exceeds the minimal clinical important difference of 17.5% suggested by Button et al.^52^ Improvement in quality of life was also observed, with the reduction in PDQ-39-SI scores at endpoint and 3-months follow-up exceeding the minimal clinically important difference of −4.72 points suggested by Horváth and colleagues.^40^ IPRI subscale scores improved in the expected direction following acute treatment, although the amount of change was modest relative to the other measures. Nevertheless, at endpoint, mean social support scores were higher and mean conflict scores were lower than normative data derived from other patient populations and the general population in the US^41^ and Australia.^53^

Not surprisingly, the primary focus of IPT for most patients was role transitions, which involved difficulty adapting to the loss of the healthy self, progressive loss of physical functioning, loss of valued social roles that were central to their identity and self-esteem, and other significant life changes. For many, changes in physical functioning decreased activities in daily living, shifting dependency on others and diminishing their sense of competence and autonomy. IPT acknowledges the reality of loss and immense burden faced by patients with serious medical diseases and helps them mourn the losses, cope with the fears of further losses, and then move forward and make the most of their life.^49,50^ A brief vignette that illustrates the application of IPT for role transitions in PD is provided in the Appendix. Role dispute was a secondary focus of therapy in two patients and revolved around strained relationships with a spouse and children. The nature of the disputes was complex and involved frequent negative exchanges about unmet expectations, feeling disrespected and misunderstood, and feeling neglected and taken advantage of. While these relationship struggles preceded the onset of disease and are not unique to patients with PD, the illness added an additional layer of strain to an already fragile relationship, leaving the patient feeling discouraged that things could improve. The treatment of role disputes involved helping patients better understand the nature of the dispute, exploring options for changing the relationship, and modifying expectations and unhelpful communication patterns.^32^ Finally, interpersonal deficit was the primary focus of one patient and secondary focus of another. These patients presented with longstanding difficulties developing and maintaining relationships. They had few supports and spent most of their time alone. The treatment of interpersonal deficits involves reducing social isolation and improving or facilitating the development of social ties.^32^ People with interpersonal deficits have been found to be less responsive to IPT^13^ and in the current study both patients with interpersonal deficits were classified as non-responders. It is likely that 12 sessions of IPT is not an adequate dose for patients who present with interpersonal deficits and a longer course of therapy may be needed.

The impact PD had on close relationships was addressed in therapy irrespective of focal area. Chronic disease and loss of autonomy and physical functioning inevitably alters the dynamics of relationships. Although most patients in this study felt adequately supported by others, they were preoccupied with being a burden, reluctant to express their needs and frustrations with others because they believed they were in a subordinate position, and avoided disclosing their illness to others, including close relatives, for fear of distressing them or being pitied. The visible signs of PD, slowing of cognitive processes, and speech and voice problems were also difficult to bear for some patients and contributed to diminished enjoyment in usual social interactions and avoidance. Fear of rejection and abandonment, especially by their life partner, was another preoccupation for some patients. Although adult attachment style was not assessed in this feasibility study, chronic illness can increase attachment insecurity, which in turn, contributes to adverse health and mental health outcomes.^54^ Helping patients understand the impact PD has had on important relationships, better tolerate and navigate changed relationships, and improve interpersonal skills were among the IPT strategies used to address these interpersonal ramifications of PD.

In sum, results of this feasibility study are encouraging and suggest that IPT is well-accepted and tolerated by depressed PD patients and requires minimal modifications. The study also provides preliminary evidence that IPT may improve PD patients’ mood and quality of life. However, findings should be viewed with caution as this was an open trial with a small sample size and unblinded HAM-D ratings, and we cannot ascertain if improvement was due to IPT or spontaneous improvement, placebo effect, or regression to the mean. It is also important to note that we included patients with Stage I-III PD and cannot conclude that IPT would be feasible or of benefit to patients with advanced stages of the disease who experience severely disabling motor symptoms, greater cognitive deficits, and require nursing home care.^55^ Currently, we are conducting a RCT on the efficacy of IPT for PD-depression. The results of this trial will determine more definitively whether IPT should be considered part of the treatment armamentarium for depressed Parkinson’s patients.

## Appendix 1

### Vignette: IPT for Role Transition

Note: This case is fictitious and constructed using material from several individuals. All material has been thoroughly altered to protect confidentiality.

Mr. A is a married male in his early 60s with a 5-year history of PD. In the last year his motor symptoms had worsened, and he began to experience some mild impairment in executive function and processing speed. He decided to retire earlier than intended from his position as a business analyst because it became more difficult for him to function at work and his motor symptoms worsened when he was under stress. The unplanned retirement was psychologically painful, and he became increasingly distressed about finding himself with an uncertain future, angry about the unwanted changes in his life brought about by PD and terrified of being placed in a long-term care facility. For the past several months he experienced symptoms consistent with a diagnosis of major depression; this included sadness, loss of pleasure, low energy, disturbed sleep and appetite, and feelings of worthlessness. He was socially withdrawn, passive, and felt guilty about not doing more and helping his wife. He had no previous history of depression or contact with a mental health care provider. Mr. A accepted that he was depressed but felt anyone would be given his circumstances. He was provided psychoeducation about depression in the context of PD, informed that clinical depression was an illness and not his fault or a “normal” reaction to living with PD, reassured that his depression was a treatable condition, and encouraged to try his best to keep active in daily activities.

A review of Mr. A’s interpersonal inventory revealed that he had a good network of social supports and history of forming enduring attachments. He had a satisfying relationship with his wife of 28 years. She was supportive and rarely complained about the added responsibilities at home. Mr. A’s wife was very troubled by the PD diagnosis, and she still struggled to accept it. Although they had always been close and enjoyed each other’s company, he felt their relationship had changed in subtle ways since his diagnosis. Most distressing to him was his wife’s greater distance, resulting in feelings of self-doubt and insecurity. Mr. A avoided talking to his wife about how difficult things had been for him in the last year because he did not want to upset her. He felt guilty about the PD diagnosis and how the disease had altered and will continue to alter her life. Although he was still self-sufficient, he worried a great deal about her ability to cope as the disease progressed. Mr. A described a good relationship with his daughter who lived and worked abroad. They video chatted once a week and this helped brighten his mood. Mr. A was very protective of his daughter, and he kept their conversations positive and avoided discussing details of his illness. Mr. A had three close friends he had known since childhood. He used to socialize with them once a week, but the frequency of contact decreased, in part because of the unpredictability of his PD symptoms and because he often had little energy to do things. He appreciated his friends’ efforts to adjust their activities to accommodate the PD and offers to help him with home repairs, but this only made him feel embarrassed and like a burden. Mr. A had two older siblings who lived in nearby towns and a large extended family he would see at family gatherings. He hid his depression from his siblings but knew he could count on them for support if needed. They worried that he was pulling away from the family and encouraged him to attend family gatherings. However social interactions had become more effortful and less satisfying and his participation in family events gradually diminished. He did not report recent deaths of relatives or close friends. Both of his parents and an older sibling died several years ago and although he would often think about them and missed them, there was no evidence of pathological grief.

After reviewing the interpersonal and social context of Mr. A’s depression it was clear that his depression was linked to the deterioration of his PD and loss of important social roles and valued activities. Despite the availability of supports, Mr. A had difficulty accepting help from others and was reticent to discuss his current struggles and express his needs. The therapist formulated the case as a role transition and treatment goals were collaboratively set and included helping Mr. A process the painful losses associated with declining health and unplanned retirement, adjust to his new circumstances, strengthen the bond with his wife, and increase his level of comfort seeking out and receiving support from others.

In the middle phase of therapy, Mr. A was asked to describe what his life was like prior to the PD diagnosis, including positive and negative aspects, and how his life had changed since the diagnosis. He described a satisfying work, family, and social life prior to becoming ill and he was active in his community. Since the onset of PD symptoms, he had experienced an accumulation of big and little losses, and he had gradually lost confidence in himself and felt lost. He missed being professionally active as well as the social contacts from work and outings with family and friends. Mr. A was encouraged to share his feelings about the losses with the therapist who validated them and acknowledged that PD was a challenging disease to live with. Talking about painful emotions provided Mr. A with an unexpected sense of relief because he rarely discussed his struggles with others, in part because he did not want people to worry about him or feel sorry for him, and because it was important for him to show others that he was stoic in the face of adversity and able to cope alone. Mr. A admitted that keeping up a good front with others was exhausting and not eliciting support from those he felt closest to only left him feeling alone in his suffering. As Mr. A described his unplanned retirement, he acknowledged that his work had dominated his life, leaving little time for him to travel and pursue other interests. The therapist suggested that perhaps retiring opened an opportunity for him to do things he never had time to do, and that he should seize the chance to travel while he was still able to walk without too much difficulty. Further sessions helped Mr. A clarify what his new priorities in life were and explore valued activities he could adapt so he could continue doing them and remain engaged in life and connected to others.

As Mr. A became less entangled in loss and capable of experiencing more positive emotions, he began to feel more confident in his ability to navigate the changes in health and social roles and optimistic that he could still have a meaningful life despite his current limitations and uncertain future. He gradually became less withdrawn and re-engaged in some activities that were not too taxing for him. Although he still felt uncomfortable sharing his feelings with others, he was more receptive to seeking and receiving support but felt it was important for him to outline boundaries so he would not be perceived as a burden or a responsibility. The therapist and Mr. A continued to talk about his wife’s struggles with his diagnosis and his sense that she had become more distant. He was encouraged to selectively communicate his feelings, needs, and wishes to her, and develop a better understanding of her needs and experiences living with a spouse with PD. For example, the therapist and Mr. A role-played how he might communicate his wish for greater closeness, and they discussed ways he could check in on his wife to let her express her own struggles and help her feel supported. Mr. A noted that his wife appreciated his efforts to be supportive and he found that sharing some of his feelings increased their connection to each other. He added that he and his wife decided that it would be most helpful for them to focus on the here and now and the positive aspects of their lives, rather than worrying excessively about the future, and engage in activities they could both enjoy and that would nourish their relationship.

In final stage of therapy, the therapist and Mr. A talked about the gains he had made in treatment, maintenance of treatment gains, relapse prevention and feelings about ending therapy. Mr. A felt he was more in control of his life and although he continued to have periods of sadness, he was no longer clinically depressed, and his social functioning had improved. He was slightly anxious about ending therapy but did not feel additional sessions were needed. Mr. A and the therapist agreed to meet in a few months to “check-in.”
